# *N*-(3-Chloro-2-methyl­phen­yl)-6-oxo-1,6-di­hydro­pyridine-3-carboxamide

**DOI:** 10.1107/S2414314623006028

**Published:** 2023-07-14

**Authors:** Guimiao Tian, Sihui Long

**Affiliations:** a School of Chemical Engineering and Pharmacy, Wuhan Institute of Technology, Wuhan, Hubei 430205, People’s Republic of China; Katholieke Universiteit Leuven, Belgium

**Keywords:** crystal structure, 6-oxo-1,6-di­hydro­pyridine, N—H⋯O hydrogen bonds

## Abstract

In the crystal structure of the title compound, C_13_H_11_ClN_2_O_2_, the mol­ecules form a three-dimensional network based on two types of hydrogen bonds between NH groups and the carbonyl oxygen atoms and amides. The mol­ecule is highly twisted, as evidenced by the dihedral angle between the 6-oxo-1,6-di­hydro­pyridine and benzene rings [88.1 (2)°].

## Abstract 2

The title compound was synthesized with 6-oxo-1,6-di­hydro­pyridine-3-carb­oxy­lic acid and 3-chloro-2-methyl­aniline as starting materials. The crystal packing is characterized by two types of N—H⋯O hydrogen bonds.

## Graphical abstract

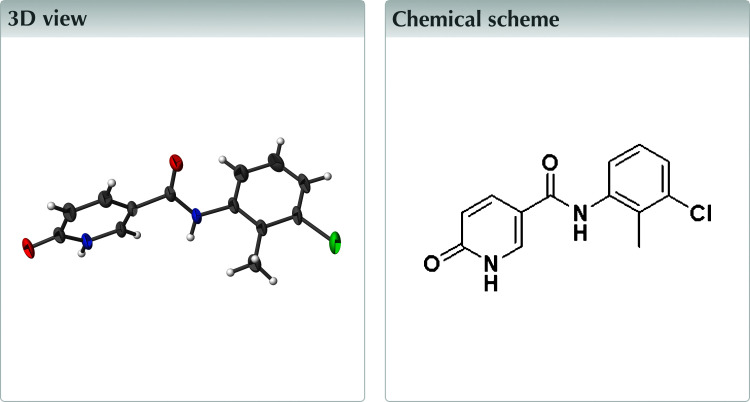

## Structure description

**Figure 1:**
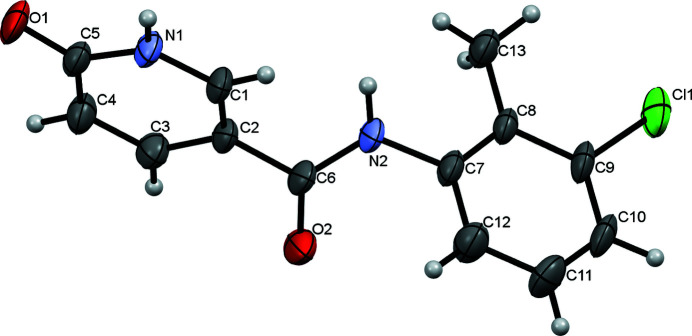
Mol­ecular structure of the title compound, with displacement ellipsoids drawn at the 50% probability level.

**Figure 2:**
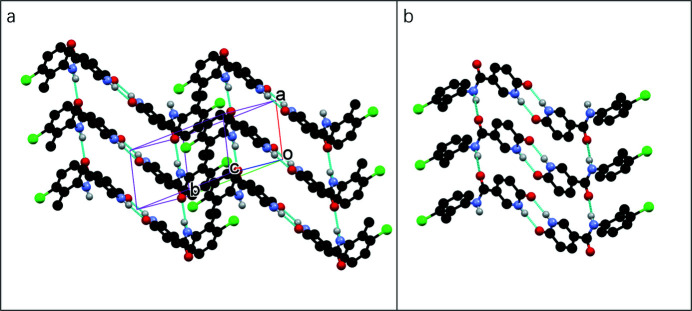
(*a*) Packing of the mol­ecules in the title compound; (*b*) chain and dimer formation supported by inter­molecular N–H⋯O=C hydrogen bonds (indicated by blue dashed lines).

The title compound (Fig. 1 ▸) is a structural isomer of *N*-phenyl-2-hy­droxy­nicotinanilide, which has inter­esting structural properties (Liu *et al.*, 2020 ▸; Zhoujin *et al.*, 2021 ▸). We wondered if isomerization would lead to completely different synthons in the crystal structure. In our study, crystals were obtained by slowly evaporating a pyridine solution of the title compound. The mol­ecule is highly twisted, as evidenced by the dihedral angle between the 6-oxo-1,6-di­hydro­pyridine and benzene rings [88.1 (2)°]. In the crystal, the mol­ecules form chains running in the *a*-axis direction through hydrogen bonds between NH groups and the carbonyl oxygen atoms of the amides (Fig. 2 ▸, Table 1 ▸). The 6-oxo-1,6-di­hydro­pyridine rings form dimers through additional N—H⋯O hydrogen bonds.

## Synthesis and crystallization

**Figure 3:**
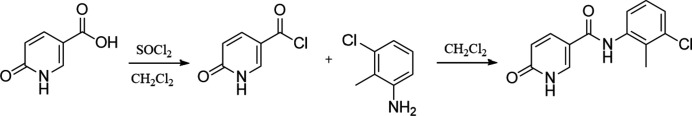
Synthesis of the title compound.

**Figure 4:**
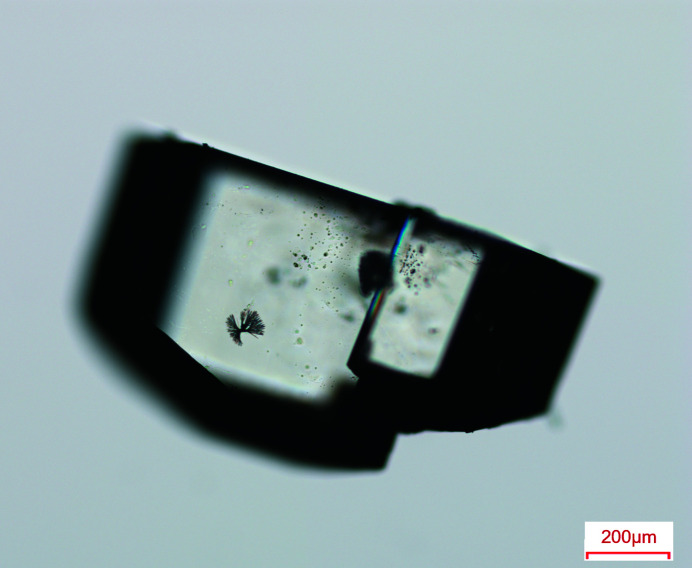
A representative crystal of the title compound.

The title compound was synthesized with 6-oxo-1,6-di­hydro­pyridine-3-carb­oxy­lic acid and 3-chloro-2-methyl­aniline as starting materials (Fig. 3 ▸). The pure sample was dissolved in bulk pyridine at 323 K, and the resulting solution was left in a refrigerator. Colorless block-shaped crystals (Fig. 4 ▸) were harvested after several days.

## Refinement

Crystal, data collection and refinement details are presented in Table 2 ▸.

## Supplementary Material

Crystal structure: contains datablock(s) global, I. DOI: 10.1107/S2414314623006028/vm4061sup1.cif

Structure factors: contains datablock(s) I. DOI: 10.1107/S2414314623006028/vm4061Isup2.hkl

Supporting information file. DOI: 10.1107/S2414314623006028/vm4061Isup3.cml

CCDC reference: 2280200

Additional supporting information:  crystallographic information; 3D view; checkCIF report

## Figures and Tables

**Table 1:** Hydrogen-bond geometry (Å, °)

*D*—H⋯*A*	*D*—H	H⋯*A*	*D*⋯*A*	*D*—H⋯*A*
N1—H1⋯O1^i^	0.86	1.93	2.793 (3)	177
N2—H2⋯O2^ii^	0.86	2.08	2.926 (3)	166

Symmetry codes: (i) 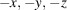 ; (ii) 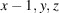 .

**Table 2:** Experimental details

Crystal data	
Chemical formula	C_13_H_11_ClN_2_O_2_
*M*_r_	262.69
Crystal system, space group	Triclinic, *P* 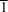
Temperature (K)	268
*a*, *b*, *c* (Å)	4.91237 (15), 10.3037 (3), 12.5876 (3)
α, β, γ (°)	105.890 (2), 96.422 (2), 99.361 (2)
*V* (Å^3^)	596.35 (3)
*Z*	2
Radiation type	Cu *K*α
μ (mm^−1^)	2.81
Crystal size (mm)	0.11 × 0.05 × 0.04

Data collection	
Diffractometer	Rigaku Oxford Diffraction, Synergy Custom system, HyPix
Absorption correction	Multi-scan (*CrysAlis PRO*; Rigaku OD, 2021 ▸)
*T*_min_, *T*_max_	0.482, 1.000
No. of measured, independent and observed [*I* > 2σ(*I*)] reflections	5870, 2362, 2023
*R*_int_	0.059
(sin θ/λ)_max_ (Å^−1^)	0.633

Refinement	
*R*[*F* ^2^ > 2σ(*F* ^2^)], *wR*(*F* ^2^), *S*	0.060, 0.184, 1.09
No. of reflections	2362
No. of parameters	164
H-atom treatment	H-atom parameters constrained
Δρ_max_, Δρ_min_ (e Å^−3^)	0.34, −0.47

Computer programs: *CrysAlis PRO* (Rigaku OD, 2021 ▸), *SHELXT* (Sheldrick, 2015*a* ▸), *SHELXL* (Sheldrick, 2015*b* ▸), *OLEX2* (Dolomanov *et al.*, 2009 ▸) and *Mercury* (Macrae *et al.*, 2020 ▸).

## full crystallographic data

### Crystal data

**Table:**

C_13_H_11_ClN_2_O_2_	*Z* = 2
*M**_r_* = 262.69	*F*(000) = 272
Triclinic, *P*1	*D*_x_ = 1.463 Mg m^−^^3^
*a* = 4.91237 (15) Å	Cu *K*α radiation, λ = 1.54184 Å
*b* = 10.3037 (3) Å	Cell parameters from 4316 reflections
*c* = 12.5876 (3) Å	θ = 3.7–77.4°
α = 105.890 (2)°	µ = 2.81 mm^−^^1^
β = 96.422 (2)°	*T* = 268 K
γ = 99.361 (2)°	Block, clear light colourless
*V* = 596.35 (3) Å^3^	0.11 × 0.05 × 0.04 mm

### Data collection

**Table:**

Rigaku Oxford Diffraction, Synergy Custom system, HyPix diffractometer	2362 independent reflections
Radiation source: Rotating-anode X-ray tube, Rigaku (Cu) X-ray Source	2023 reflections with *I* > 2σ(*I*)
Mirror monochromator	*R*_int_ = 0.059
Detector resolution: 10.0000 pixels mm^-1^	θ_max_ = 77.6°, θ_min_ = 3.7°
ω scans	*h* = −6→6
Absorption correction: multi-scan (CrysAlisPro; Rigaku OD, 2021)	*k* = −12→12
*T*_min_ = 0.482, *T*_max_ = 1.000	*l* = −15→11
5870 measured reflections	

### Refinement

**Table:**

Refinement on *F*^2^	Primary atom site location: dual
Least-squares matrix: full	Hydrogen site location: inferred from neighbouring sites
*R*[*F*^2^ > 2σ(*F*^2^)] = 0.060	H-atom parameters constrained
*wR*(*F*^2^) = 0.184	*w* = 1/[σ^2^(*F*_o_^2^) + (0.1286*P*)^2^ + 0.0079*P*] where *P* = (*F*_o_^2^ + 2*F*_c_^2^)/3
*S* = 1.09	(Δ/σ)_max_ < 0.001
2362 reflections	Δρ_max_ = 0.34 e Å^−^^3^
164 parameters	Δρ_min_ = −0.47 e Å^−^^3^
0 restraints	

### Special details

**Table:**

Geometry. All esds (except the esd in the dihedral angle between two l.s. planes) are estimated using the full covariance matrix. The cell esds are taken into account individually in the estimation of esds in distances, angles and torsion angles; correlations between esds in cell parameters are only used when they are defined by crystal symmetry. An approximate (isotropic) treatment of cell esds is used for estimating esds involving l.s. planes.
Refinement. positions of H atoms at N1 and N2 were obtained from a difference Fourier map. Other H atoms were positioned geometrically with C—H = 0.93 Å (aromatic H) or 0.96 Å (methyl H), and constrained to ride on their parent atoms, with *U*_iso_(H) = *xU*_eq_(C,*O*), where *x*=1.5 for methyl H, and *x*=1.2 for all other H atoms.

### Fractional atomic coordinates and isotropic or equivalent isotropic displacement parameters (Å^2^)

**Table:**

	*x*	*y*	*z*	*U*_iso_*/*U*_eq_	
Cl1	0.70169 (15)	0.48597 (7)	0.88602 (4)	0.0568 (3)	
O1	0.2394 (4)	0.10879 (18)	−0.04783 (12)	0.0467 (4)	
O2	1.0545 (3)	0.2472 (2)	0.40507 (14)	0.0554 (5)	
N1	0.2702 (4)	0.07879 (18)	0.12455 (14)	0.0351 (4)	
H1	0.113762	0.020388	0.103199	0.042*	
N2	0.6373 (4)	0.23089 (19)	0.46700 (13)	0.0367 (4)	
H2	0.459644	0.221023	0.448300	0.044*	
C1	0.3982 (4)	0.1067 (2)	0.23115 (16)	0.0344 (5)	
H1A	0.313595	0.064270	0.278844	0.041*	
C2	0.6488 (4)	0.1959 (2)	0.27013 (16)	0.0327 (4)	
C3	0.7723 (5)	0.2563 (2)	0.19387 (18)	0.0387 (5)	
H3	0.946826	0.314972	0.217320	0.046*	
C4	0.6407 (5)	0.2301 (2)	0.08749 (18)	0.0403 (5)	
H4	0.724547	0.272452	0.039606	0.048*	
C5	0.3741 (4)	0.1379 (2)	0.04762 (16)	0.0338 (5)	
C6	0.7985 (4)	0.2265 (2)	0.38631 (17)	0.0357 (5)	
C7	0.7463 (4)	0.2510 (2)	0.58157 (16)	0.0330 (5)	
C8	0.6758 (4)	0.3534 (2)	0.66538 (16)	0.0312 (4)	
C9	0.7869 (5)	0.3634 (2)	0.77585 (17)	0.0369 (5)	
C10	0.9636 (5)	0.2817 (3)	0.80184 (19)	0.0436 (5)	
H10	1.034339	0.292535	0.876128	0.052*	
C11	1.0340 (6)	0.1845 (3)	0.7173 (2)	0.0498 (6)	
H11	1.156189	0.129966	0.733910	0.060*	
C12	0.9234 (6)	0.1669 (2)	0.6069 (2)	0.0465 (6)	
H12	0.967423	0.098984	0.549550	0.056*	
C13	0.4974 (5)	0.4497 (2)	0.64070 (18)	0.0413 (5)	
H13A	0.614874	0.532952	0.638879	0.062*	
H13B	0.384069	0.471175	0.697991	0.062*	
H13C	0.378964	0.406824	0.569413	0.062*	

### Atomic displacement parameters (Å^2^)

**Table:**

	*U*^11^	*U*^22^	*U*^33^	*U*^12^	*U*^13^	*U*^23^
Cl1	0.0737 (5)	0.0721 (5)	0.0188 (4)	0.0169 (3)	0.0033 (3)	0.0046 (3)
O1	0.0527 (10)	0.0628 (10)	0.0219 (8)	0.0063 (7)	−0.0090 (7)	0.0172 (7)
O2	0.0327 (9)	0.1011 (14)	0.0257 (9)	0.0141 (8)	−0.0021 (6)	0.0103 (9)
N1	0.0348 (9)	0.0473 (9)	0.0185 (9)	0.0040 (7)	−0.0040 (6)	0.0080 (7)
N2	0.0337 (9)	0.0570 (10)	0.0151 (9)	0.0098 (7)	−0.0028 (6)	0.0060 (7)
C1	0.0374 (11)	0.0466 (10)	0.0172 (10)	0.0085 (8)	0.0000 (8)	0.0078 (8)
C2	0.0349 (10)	0.0440 (10)	0.0158 (9)	0.0105 (8)	−0.0012 (7)	0.0037 (8)
C3	0.0354 (11)	0.0483 (11)	0.0284 (11)	0.0051 (8)	0.0008 (8)	0.0087 (9)
C4	0.0455 (12)	0.0502 (11)	0.0258 (11)	0.0050 (9)	0.0024 (9)	0.0162 (9)
C5	0.0400 (11)	0.0406 (10)	0.0211 (10)	0.0116 (8)	−0.0009 (8)	0.0101 (8)
C6	0.0344 (11)	0.0491 (11)	0.0186 (10)	0.0098 (8)	−0.0036 (8)	0.0045 (8)
C7	0.0355 (10)	0.0430 (10)	0.0184 (10)	0.0052 (8)	−0.0026 (7)	0.0102 (8)
C8	0.0344 (10)	0.0409 (10)	0.0181 (10)	0.0031 (7)	0.0002 (7)	0.0125 (8)
C9	0.0455 (12)	0.0473 (11)	0.0155 (10)	0.0026 (8)	−0.0009 (8)	0.0116 (8)
C10	0.0500 (13)	0.0597 (13)	0.0229 (11)	0.0053 (10)	−0.0048 (9)	0.0227 (10)
C11	0.0577 (15)	0.0569 (13)	0.0415 (14)	0.0171 (11)	−0.0031 (11)	0.0269 (12)
C12	0.0582 (15)	0.0492 (12)	0.0333 (13)	0.0196 (10)	0.0010 (10)	0.0117 (10)
C13	0.0504 (13)	0.0490 (11)	0.0247 (11)	0.0157 (9)	−0.0013 (9)	0.0114 (9)

### Geometric parameters (Å, º)

**Table:**

Cl1—C9	1.743 (2)	C3—C4	1.355 (3)
O1—C5	1.238 (2)	C4—C5	1.434 (3)
O2—C6	1.225 (3)	C7—C8	1.391 (3)
N1—C1	1.349 (3)	C7—C12	1.394 (3)
N1—C5	1.379 (3)	C8—C9	1.406 (3)
N2—C6	1.351 (3)	C8—C13	1.497 (3)
N2—C7	1.427 (2)	C9—C10	1.376 (3)
C1—C2	1.360 (3)	C10—C11	1.366 (4)
C2—C3	1.418 (3)	C11—C12	1.387 (3)
C2—C6	1.487 (3)		
			
C1—N1—C5	124.19 (18)	N2—C6—C2	116.39 (17)
C6—N2—C7	123.50 (17)	C8—C7—N2	119.96 (17)
N1—C1—C2	121.18 (19)	C8—C7—C12	121.34 (18)
C1—C2—C3	117.44 (18)	C12—C7—N2	118.70 (19)
C1—C2—C6	122.53 (18)	C7—C8—C9	116.00 (18)
C3—C2—C6	119.98 (18)	C7—C8—C13	122.62 (17)
C4—C3—C2	121.2 (2)	C9—C8—C13	121.36 (19)
C3—C4—C5	120.99 (19)	C8—C9—Cl1	118.95 (17)
O1—C5—N1	119.92 (19)	C10—C9—Cl1	117.88 (16)
O1—C5—C4	125.14 (19)	C10—C9—C8	123.2 (2)
N1—C5—C4	114.93 (17)	C11—C10—C9	119.3 (2)
O2—C6—N2	123.42 (19)	C10—C11—C12	120.1 (2)
O2—C6—C2	120.18 (19)	C11—C12—C7	120.1 (2)

### Hydrogen-bond geometry (Å, º)

**Table:**

*D*—H···*A*	*D*—H	H···*A*	*D*···*A*	*D*—H···*A*
N1—H1···O1^i^	0.86	1.93	2.793 (3)	177
N2—H2···O2^ii^	0.86	2.08	2.926 (3)	166

Symmetry codes: (i) −*x*, −*y*, −*z*; (ii) *x*−1, *y*, *z*.
